# Clinical Utility of Frailty Scoring in Elderly Acute Myeloid Leukemia Patients Treated With Venetoclax and Hypomethylating Agents

**DOI:** 10.1111/ejh.70058

**Published:** 2025-10-29

**Authors:** Ernesto Vigna, Antonella Bruzzese, Enrica Antonia Martino, Andrea Corsonello, Santino Caserta, Caterina Labanca, Francesco Mendicino, Eugenio Lucia, Virginia Olivito, Nicola Amodio, Fortunato Morabito, Massimo Gentile

**Affiliations:** ^1^ Hematology Unit, Department of Onco‐Hematology AO of Cosenza Cosenza Italy; ^2^ Unit of Geriatric Medicine Italian National Research Center on Aging (IRCCS INRCA) Cosenza Italy; ^3^ Department of Pharmacy, Health and Nutritional Science University of Calabria Rende Italy; ^4^ Department of Experimental and Clinical Medicine University of Catanzaro Catanzaro Italy; ^5^ AIL Sezione di Cosenza Cosenza Italy

**Keywords:** AML, frailty, therapy

## Abstract

Acute myeloid leukemia (AML) in elderly patients presents a major therapeutic challenge, as many are deemed unfit for intensive chemotherapy due to age, comorbidities, or frailty. Venetoclax in combination with hypomethylating agents (HMA) has emerged as a standard‐of‐care for this population, yet outcomes remain heterogeneous and predictive tools are limited. In this retrospective single‐center study, we analyzed 52 treatment‐naïve AML patients receiving venetoclax combined with either azacitidine (*n* = 33) or decitabine (*n* = 19) at the Hematology Department of Cosenza Hospital between August 2021 and June 2025. Frailty was assessed using the Clinical Frailty Scale (CSHA CFS), with 19 patients classified as low frailty (score ≤ 3) and 33 as high frailty (score > 3). The median age was 75.3 years (range 58.2–89.2), and the cohort included 33 de novo and 19 secondary AML cases. After a median follow‐up of 18 months, 34 patients (65.4%) had died: 14 due to disease progression, 12 due to treatment‐related toxicity—predominantly severe infections—and 5 from unrelated causes. ROC curve analysis showed that a CFS score > 3 was associated with worse survival (AUC 0.75, 95% CI 0.61–0.89, *p* < 0.004), with median overall survival of 7.6 months for low‐frailty patients versus 2.5 months for high‐frailty patients (1‐year OS 44.1% vs. 18.7%, *p* = 0.031). Multivariate analysis confirmed that lower frailty (*p* = 0.031) and azacitidine‐based therapy (*p* = 0.025) were independently associated with improved survival. Overall response rate was 48%, including 21 complete responses (CR/CRi) and 4 partial responses. Frailty was the only significant predictor of response (*p* = 0.005), whereas age, sex, type of AML, ELN risk score, renal function, BMI, or type of HMA did not significantly influence outcomes. Grade 3–4 treatment‐related adverse events occurred in all patients, predominantly hematological; non‐hematological events included infections (61.9%), cardiotoxicity (11.9%), and liver toxicity (9.5%). High‐frailty patients experienced a higher incidence of infections (72.7% vs. 36.8%, *p* = 0.02) and hospitalizations (57.6% vs. 21.1%, *p* = 0.011). These results suggest that the CSHA CFS is a simple and clinically meaningful tool to stratify elderly AML patients for venetoclax–HMA therapy, identifying those at higher risk of treatment‐related complications and poor survival. Incorporating frailty assessment into routine practice may enhance patient selection, optimize supportive care, and guide individualized therapeutic decisions. Prospective, multicenter studies are warranted to validate these findings and refine the use of frailty‐guided treatment strategies in this vulnerable population.

## 1 Introduction

Acute myeloid leukemia (AML) predominantly affects older adults, and a substantial proportion of newly diagnosed patients are deemed unfit for intensive chemotherapy due to advanced age, comorbidities, or frailty [1]. Historically, these patients faced poor outcomes and limited therapeutic options, including hypomethylating agents (HMAs), both azacitidine (AZA) and decitabine (DEC), and low‐dose cytarabine [2]. However, recent advances in the understanding of AML biology and the development of targeted agents have transformed the management landscape for this vulnerable group reviewed in [3].

The combination of the BCL2 inhibitor venetoclax with an HMA has emerged as the standard‐of‐care for most unfit patients, providing higher response rates and survival compared to earlier low‐intensity regimens [4, 5, 6, 7], while also delaying deterioration in quality of life [8]. In addition, the availability of oral small molecule inhibitors targeting FLT3, IDH1/2, and menin (for NPM1‐mutated or KMT2A‐rearranged AML) has further broadened the therapeutic armamentarium [1]. As a result, treatment selection now requires a nuanced, individualized approach that incorporates not only disease genetics but also patient fitness, quality‐of‐life considerations, and personal preferences [9, 10].

Despite these advances, the 5‐year overall survival (OS) of elderly AML patients treated with HMA‐based therapies remains unsatisfactory, ranging from 5% to 10% [11, 12].

In recent years, increasing attention has been focused not only on developing more effective therapies but also on optimizing the management of frail patients to minimize treatment‐related toxicity.

The European Leukaemia Net (ELN) recommends several strategies for patients unfit for intensive treatment and emphasizes the need for better fitness stratification based on both patient‐ and disease‐related factors [13].

Assessing fitness and comorbidities is therefore a crucial component of the diagnostic workup for all patients, as it guides therapeutic intensity [14, 15, 16, 17, 18].

Comprehensive geriatric assessment, evaluating clinical, cognitive, physical, and social domains, has been investigated as a prognostic tool in elderly AML, but its complexity limits routine applicability, and no consensus exists on its standardized use [19, 20, 21].

To overcome these limitations, simplified tools have been proposed, sometimes integrating disease‐related factors such as cytogenetic and molecular abnormalities (e.g., AML Composite model, AML‐CM) [22, 23, 24, 25].

While such scores have improved fitness assessment, their prognostic significance in AML patients receiving non‐intensive therapy remains uncertain, largely due to limited validation in real‐world settings.

The Canadian Study of Health and Aging (CSHA) Clinical Frailty Scale (CFS) is a simple, clinically applicable tool that classifies older individuals based on vulnerability and risk of death. Originally developed to predict early mortality among elderly patients in the emergency department, it has since been extended to other medical contexts, including oncology and hematology [26]. However, its role has never been specifically investigated in AML [27, 28, 29].

In this study, we analysed both disease‐ and patient‐related factors, including frailty as measured by CSHA CFS, to evaluate their ability to predict outcomes in AML patients treated with HMAs and venetoclax.

## 2 Materials and Methods

### 2.1 Study Design

We conducted a retrospective single‐center study to evaluate the efficacy of HMAs (AZA or DEC) in combination with VEN in treatment‐naïve (TN) AML patients deemed unfit for intensive therapy due to age or comorbidities. Patients were treated at the Hematology department of Cosenza Hospital.

The primary aim was to analyse OS in the entire cohort and compare outcomes according to the CFS [26]. We used the 7‐point CFS version (Table 1). Each category provides a clinical description, ranging from “Very fit”—indicating robust, active, and energetic individuals who commonly exercise regularly—to “Severely frail,” indicating complete dependence on others or terminal illness. Intermediate categories include: “Well”, describing individuals free of active disease but less fit than the very fit; “Well, with treated comorbid disease” for those with well‐controlled symptoms; “Apparently vulnerable” for individuals experiencing slowing or symptoms without dependency; “Mildly frail”, requiring help with instrumental activities of daily living; “Moderately frail”, needing assistance with both instrumental and non‐instrumental activities, and finally “Severely frail” (Table 1).

**TABLE 1:** The CSHA Clinical Frailty Scale.

Very fit—robust, active, energetic, well‐motivated and fit; these people commonly exercise regularly and are in the most fit group for their age
2 Well—without active disease, but less fit than people in category 1
3 Well, with treated comorbid disease—disease symptoms are well controlled compared with those in category 4
4 Apparently vulnerable—although not frankly dependent, these people commonly complain of being “slowed up” or having disease symptoms
5 Mildly frail—with limited dependence on others for instrumental activities of daily living
6 Moderately frail—help is needed with both instrumental and non‐instrumental activities of daily living
7 Severely frail—completely dependent on others for the activities of daily living, or terminally ill

Abbreviation: CSHA, Canadian Study of Health and Aging.

Secondary end‐points were: (i) the overall response rate (ORR) at any time during treatment, (ii) incidence of non‐hematological grade 3–4 treatment‐related adverse events (TRAEs), and (iii) the impact of patient‐ and disease‐related factors (sex, AML type, ELN 2017 risk category, body mass index [BMI], glomerular filtration rate [GFR], type of HMA, and frailty score according to CSF and CCI) on ORR and TRAEs.

The study was approved by the Institutional Ethics Committee of Catanzaro and conducted in accordance with the Declaration of Helsinki and Good Clinical Practice. Written informed consent was obtained from all patients. All data were collected from electronic health records.

### 2.2 Patient Selection

Patients were included in the present analysis if they had a confirmed diagnosis of AML according to the 2016 WHO classification (for cases diagnosed before July 2022) or the 2022 WHO classification (for those diagnosed thereafter) [12, 30].

Molecular profiling was performed using real‐time PCR and included assessment of *FLT3*, *NPM1*, *IDH1*/2, *RUNX1/RUNXT1*, and *CBFB/MYH11*. Cytogenetic analysis was performed using conventional karyotyping and FISH for del(5q), +8, del(20), del(7), and del(17p13). The CFS was applied by treating physicians at baseline. CSF was assessed at the onset and recorded for all patients.

### 2.3 Treatment Administration and Supportive Care

Selected patients had received venetoclax in combination with either azacitidine or decitabine, administered according to the approved label. Venetoclax was given orally in 28‐day cycles, with a ramp‐up schedule during cycle 1 (100 mg on Day 1, 200 mg on Day 2, and 400 mg on Day 3), followed by a target dose of 400 mg once daily thereafter. Dose adjustments were made when venetoclax was co‐administered with CYP3A inhibitors: 100 mg daily with posaconazole or other strong inhibitors, and 200 mg daily with moderate inhibitors. The venetoclax dosage was reduced after the completion of the 1st cycle according to clinical decision.

All patients received tumor lysis syndrome prophylaxis with hydration and allopurinol, or rasburicase when indicated, and laboratory monitoring was performed during the ramp‐up phase. Hospitalization was considered in high‐risk cases for close monitoring.

Azacitidine was administered subcutaneously at 75 mg/m^2^/day on days 1–7 of each 28‐day cycle. In the decitabine cohort, treatment consisted of intravenous administration at 20 mg/m^2^/day over 1 h on days 1–5 of each 28‐day cycle. Venetoclax was continued concomitantly throughout.

Concomitant supportive therapies included antimicrobial prophylaxis per institutional practice, antifungal azoles with appropriate venetoclax dose reductions, transfusion support, hydroxyurea for cytoreduction, and growth factor support for prolonged cytopenias in the absence of residual disease.

Baseline assessments included complete blood counts, serum chemistry, bone marrow morphology, flow cytometry, cytogenetics, and molecular analyses. Disease response was assessed after cycle 1 and subsequently as clinically indicated, according to European LeukemiaNet (ELN) criteria. Safety was monitored throughout treatment, with adverse events graded according to CTCAE v5.0.

Treatment was continued until disease progression, unacceptable toxicity, or transition to alternative therapy.

### 2.4 Collection of Data and Definition of Endpoints

Demographic and clinical characteristics (age, sex, GFR, BMI, Eastern Cooperative Oncology Group Performance status [ECOG‐PS]), disease features (ELN 2017 risk stratification, molecular features, type of AML), and type of HMA administered were recorded.

### 2.5 Statistical Analysis

The primary objective was to assess the prognostic value of the CFS and identify the optimal cut‐off value to predict survival. Categorical variables were compared using Fisher's exact test (for two‐way tables) or Pearson's *χ* ^2^ test (multi‐way tables). Associations between baseline variables and OS were assessed using the log‐rank test, with results presented as hazard ratios (HRs) with 95% confidence intervals (CIs). The predictive accuracy of the CFS was evaluated using receiver operating characteristic (ROC) curve analysis, with the area under the curve (AUC), sensitivity, specificity, and positive/negative predictive values, all reported with 95% CIs. The optimal cut‐off was determined using Youden's index (*J* = sensitivity + specificity −1), with the maximum value of *J* (0.243) indicating the threshold that best balances sensitivity and specificity. OS was defined as the time from treatment initiation to either the date of death or the last follow‐up, and estimated by the Kaplan–Meier method. Prognostic variables were first assessed through univariable Cox regression, with significant factors (*p* < 0.05) entered into multivariable Cox models. Definitive treatment discontinuation due to toxicity (DDT) was also recorded. All statistical analyses were performed using STATA for Windows (version 9) and SPSS Statistics (version 21).

## 3 Results

### 3.1 Patients' Characteristics

The study analysed 52 consecutive TN AML patients diagnosed at the Hematology Department of Cosenza Hospital between August 2021 and June 2025. The median age was 75.3 years (range 58.2–89.2); 19 patients were female and 33 were male. Thirty‐three patients had de novo AML, while 19 had secondary AML: 11 therapy‐related AML and 8 secondaries to other hematologic neoplasms (3 myelodysplastic syndromes, 2 non‐Hodgkin lymphomas, 1 severe aplastic anaemia, 1 chronic myelomonocytic leukemia, 1 essential thrombocytemia). Interestingly, among therapy‐related AML, 4 patients had previously received chemoimmunotherapy for lymphoproliferative disorders (2 non‐Hodgkin lymphomas, 1 chronic lymphocytic leukaemia, 1 multiple myeloma).

Nineteen patients were classified at low frailty score (≤ 3; 7 with score 1, 4 with score 2, 8 with score 3), and 33 patients as high frailty score (> 3; 8 with score 4, 11 with score 5, 8 with score 6, 5 with score 7, 1 with score 8).

At diagnosis, 8 patients had impaired renal function (GFR ≤ 30 mL/min), while 44 had normal renal function. Impaired renal function was significantly more frequent in patients with a high frailty score (24.2% vs. 0%, *p* = 0.02).

Fifteen patients were obese at diagnosis (BMI ≥ 25), while 37 had a BMI < 25. A high BMI was significantly associated with a high frailty score (39.4% vs. 10.5%, *p* = 0.027).

Considering all the comorbidities, 39 patients had a CCI < 6, while 13 had a CCI > 6.

Molecular screening by real‐time PCR was performed in 45 patients: 18 had no detectable mutations, 11 had FLT3 mutations, 9 had NPM1 mutations, 12 had IDH1/IDH2 mutations, and 3 had other mutations (2 WT1, 1 TP53).

Cytogenetic analysis was available for 37 patients: 1 had a favorable profile, 14 had an intermediate profile, and 22 had an unfavorable profile. Among the latter, 12 had a complex karyotype, 5 had monosomy 7 [2 associated with del(5q)], 1 had isolated del(5q), 3 had a trisomy 8, associated [1 associated with del(20), and 1 had isolated del(20)].

According to the ELN 2017 classification, 5 patients were categorized as favorable risk, 11 as intermediate risk, and 23 as adverse risk. Application of the ELN 2022 and 2024 criteria was not feasible due to the absence of NGS data, particularly RAS mutation testing in all patients.

Thirty‐three patients received azacitidine plus venetoclax, while 19 received decitabine plus venetoclax. The median number of treatment cycles was 4 (range 1–36). Patients' features are summarized in Table 2.

**TABLE 2:** Baseline characteristics of AML patients.

Patients' characteristics	All patients = 52	Frailty scale ≤ 3 = 19	Frailty scale > 3 = 33	*p*
Age (median)	75.3 (58.2–89.2)	74.3 (58.2–81.4)	75.7 (61.5–89.2)	0.75
Sex				
M	33	12 (63.2%)	21 (63.6%)	0.97
F	19	7 (36.8%)	12 (36.4%)	
Type of AML				
Primary	33	11 (57.9%)	22 (66.7%)	0.52
Secondary	19	8 (42.1%)	11 (33.3%)	
ELN 2017				
Favorable	5	2 (10.5%)	3 (9.1%)	
Intermediate	11	5 (26.3%)	6 (18.2%)	0.89
Unfavorable	23	8 (42.1%)	15 (45.5%)	
Not available	13	4 (21.1%)	9 (27.3%)	
GFR (ml/min)				
< 30	8	0 (0%)	8 (24.2%)	**0.02**
≥ 30	44	19 (100%)	25 (75.8%)	
BMI				
< 25	37	17 (89.5%)	20 (60.6%)	**0.027**
≥ 25	15	2 (10.5%)	13 (39.4%)	
CCI				
≤ 6	39	15 (78.9%)	24 (72.7%)	0.61
> 6	13	4 (21.1%)	9 (27.3%)	
Type of therapy				
Aza/ven	33	11 (57.9%)	22 (66.7%)	0.52
Dec/ven	19	8 (42.1%)	11 (33.3%)	

*Note:* The *p* value is statistically significant (in bold).

Abbreviations: AZA: azacitidine; BMI: body mass index; CCI: charlson comorbidity index; DEC: decitabine; ELN: European LeukemiaNet; GFR: glomerular filtration rate.

### 3.2 Overall Survival

After a median follow‐up of 18 months (range 12–24) and a median of 4 cycles (range 1–35), 34 patients (65.4%) had died. Fourteen deaths were due to disease progression, 12 to toxicity (10 of which were severe infections), 5 to unrelated causes, and in 3 cases, the cause of death was unknown.

**FIGURE 1:**
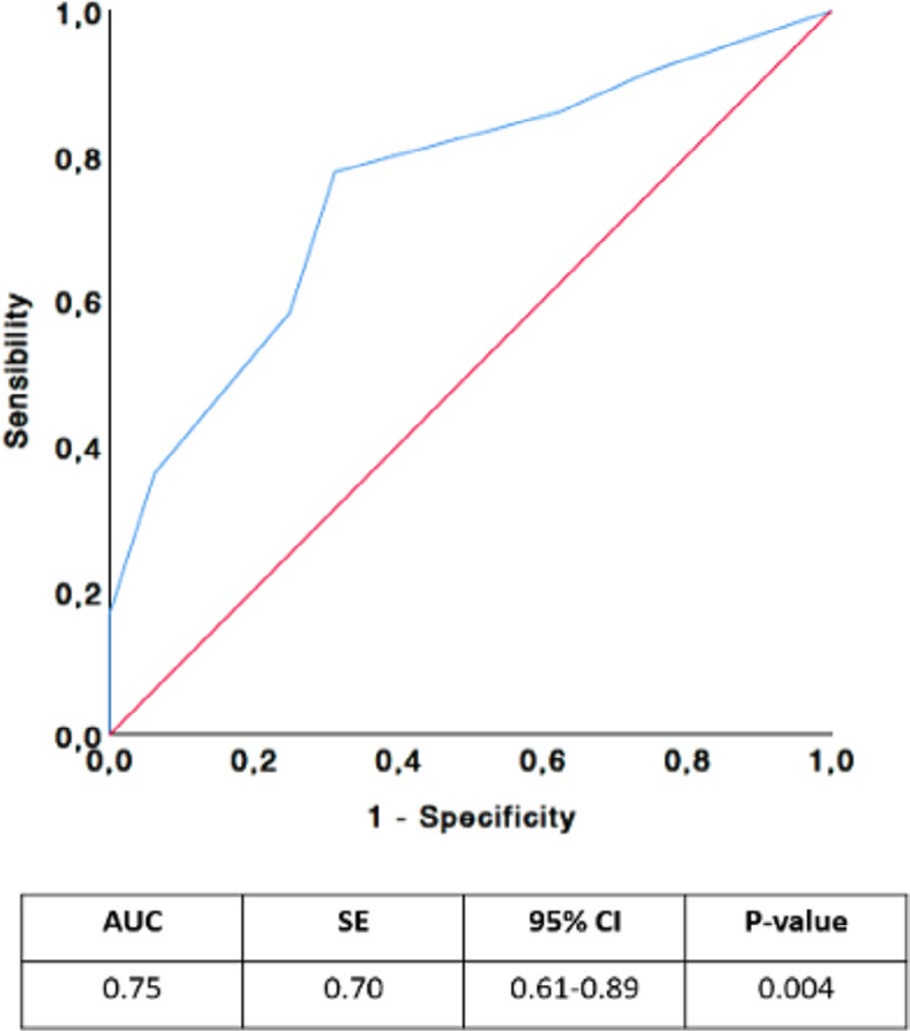
Receiver operating characteristic (ROC) analysis of Canadian Study of Health and Aging (CSHA) Clinical Frailty Scale (CFS) for predicting prognosis of AML patients treated with HMAs and venetoclax.

To evaluate the prognostic value of the frailty score for OS, ROC curve analysis was performed (Figure 1). The AUC was 0.75 (95% CI: 0.61–0.89; *p* < 0.004). The optimal cut‐off for high risk of death was a frailty score of 3, with 78% sensitivity and 69% specificity. Overall, 54 (65.9%) had a frailty score ≤ 3, and 28 patients (34.1%) had a frailty score > 3.

**FIGURE 2:**
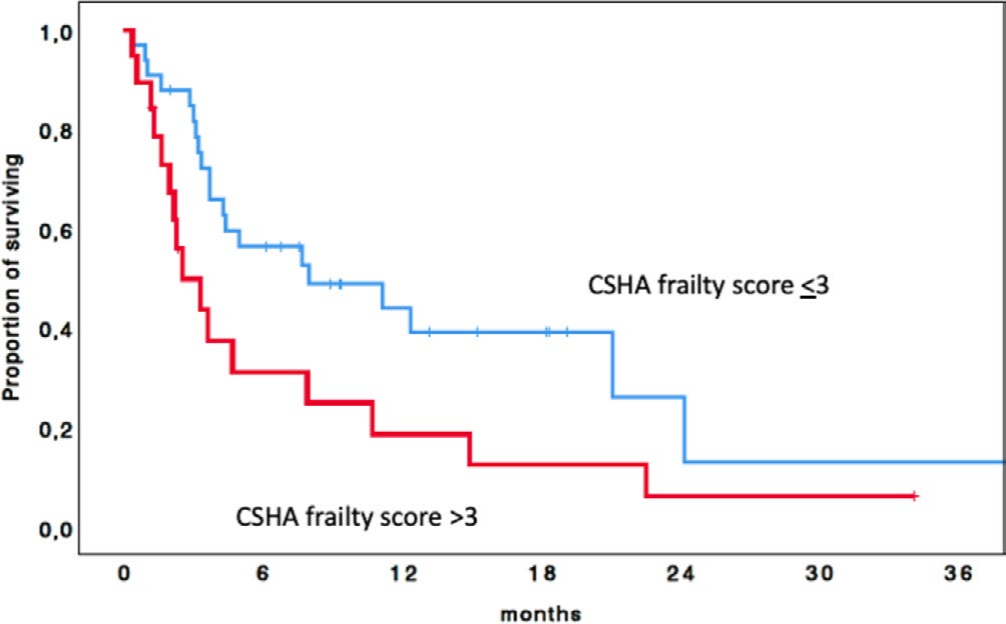
Kaplan–Meier curve of overall survival of the entire cohort according to the Canadian Study of Health and Aging (CSHA) Clinical Frailty Scale (CFS).

Median OS was significantly longer in patients with a frailty score ≤ 3 compared to > 3 (7.6 vs. 2.5 months); the 1‐year OS rates were 44.1% and 18.7%, respectively (*p* = 0.031) (Figure 2).

In multivariate analysis, lower frailty score (*p* = 0.031) and treatment with AZA/VEN vs. DEC/VEN (*p* = 0.025) were independently associated with better survival. Other variables (CCI, sex, age, type of AML, ELN risk score, GFR, BMI) showed no significant impact (Table 3).

**TABLE 3:** Univariable and multivariable analyses of overall survival.

	Univariable analyses HR (95% CI)	*p*	Multivariable analyses HR (95% CI)	*p*
Gender				
Male vs. female	1.49 (0.75–2.96)	0.25		
Age (years)				
< 75 vs. ≥ 75	1.06 (0.55–2.04)	0.87		
AML type				
Primary vs. secondary	0.92 (0.66–131)	0.67		
ELN 2017				
Intermediate vs. favorable	0.9 (0.2–3.8)	0.88		
Unfavourable vs. favorable	1.23 (0.36–4.3)	0.74		
BMI				
< 25 vs. ≥ 25	1.83 (0.92–3.63)	0.08		
GFR (ml/min)				
< 30 vs. ≥ 30	2.71 (1.16–6.35)	**0.02**	2.28 (0.93–5.59)	0.07
Type of therapy				
Aza/ven vs. Dec/ven	2.05 (1.05–3.98)	**0.035**	2.15 (1.09–4.23)	**0.025**
Frailty scale				
< 3 vs. ≥ 3	2.85 (1.28–6.3)	**0.01**	2.4 (1.05–5.5)	**0.037**
CCI				
≤ 6	2.07 (0.27–16.1)	0.49		
> 6				

*Note:* The *p* value is statistically significant (in bold).

Abbreviations: AZA: azacitidine; BMI: body mass index; CCI: charlson comorbidity index; DEC: decitabine; ELN: European LeukemiaNet; GFR: glomerular filtration rate.

### 3.3 Toxicity

The incidence of grade 3–4 TRAEs was 100%, mostly hematologic. In addition, 29 out of 42 patients (69%) experienced grade 3–4 non‐hematological TRAEs. The most frequent were infections (26 cases, 61.9%), cardiotoxicity (5 cases, 11.9%), liver toxicity (4 cases, 9.5%), stomatitis (3 cases, 7.1%), cutaneous rash (2 cases, 4.7%), respiratory distress (1 case, 2.4%), and phlebitis (1 case, 2.4%) (Table 4).

**TABLE 4:** Treatment related adverse events according to CSHA Clinical Frailty Scale.

Non haematological TRAEs	All patients = 52 (%)	Frailty scale ≤ 3 = 19 (%)	Frailty scale > 3 = 33 (%)	*p*
Infection	31 (59.6%)	7 (36.8%)	24 (72.7%)	**0.02**
Sepsis	14 (26.9%)	4 (21%)	10 (30.3%)	0.53
Gastrointestinal toxicity	14 (26.9%)	5 (26.3%)	9 (27.3%)	1.00
Cardiac toxicity	4 (7.7%)	2 (10.5%)	2 (6%)	0.61
Other	7 (13.4%)	1 (5.2%)	6 (18.2%)	0.24

*Note:* The *p* value is statistically significant (in bold).

Infections were significantly more frequent in patients with a higher frailty score (72.7% vs. 36.8%, *p* = 0.02), whereas the incidence of adverse events did not differ by frailty score.

Overall, 23 patients (44.2%) required at least one hospitalization. Among different variables (sex, age, type of AML, ELN risk group, BMI, renal function, frailty score, and type of therapy), only a high frailty score was significantly associated with the hospitalization rate (57.6% vs. 21.1%; *p* = 0.011) (Table S1).

### 3.4 Response

Twenty‐five patients achieved a response, for an ORR of 48%. One patient achieved complete response (CR) with minimal residual disease (MRD) negativity, 20 achieved CR, or CR with incomplete hematological recovery (CRi), and 4 had a partial response (PR).

The predictive capacity of baseline parameters for response was evaluated. Only the frailty score significantly predicted achievement of a response (*p* = 0.05). Conversely, neither CCI, sex, age, type of AML, type of therapy, ELN risk score, GFR, nor BMI significantly influenced response to treatment (Table S2).

## 4 Discussion

Our study shows that frailty may be helpful in predicting OS, TRAEs and ORR among older patients with AML treated with HMAs in combination with VEN. These findings suggest that frailty assessment should be considered when dealing with older patients in the hematological setting.

In recent years, with the progressive aging of the population, several treatment strategies have been developed for elderly AML patients [1]. Increasing attention has also been given to the complex management of frail patients with the goal of reducing treatment‐related toxicity. From this perspective, a reliable and practical geriatric tool is essential to guide physicians in classifying elderly patients as fit or unfit for non‐intensive treatment and in selecting among the different available therapeutic strategies.

The ELN guidelines recommend effective approaches for patients unfit for intensive chemotherapy, underlying the importance of refined fitness stratification based on both patient‐ and disease‐related factors [13].

However, most parameters used in current geriatric assessments are not easily scalable, and there is limited consensus on their routine application [18, 19, 20, 21]. Recently, a Korean retrospective study showed that an impaired short Physical Performance Battery (SPPB) score (< 10) was significantly associated with inferior OS in patients receiving HMA plus venetoclax, but not in those receiving HMAs alone, suggesting that patients with worse functional status may derive more toxicity than benefit from the addition of venetoclax [31].

Several fitness scores have been proposed for AML, particularly in predicting mortality in patients treated with intensive chemotherapy [25].

The Italian group introduced a comprehensive evaluation system to determine both eligibility for intensive therapy and suitability for non‐intensive regimens, emphasizing that elderly patients are different and are a heterogeneous population, and not all are appropriate for the same therapeutic strategies. More recently, the panel revised its criteria, considering the evolving characteristics of the geriatric population and the emergence of new therapeutic options for unfit AML patients [23, 24].

The CSHA CFS evaluates functional and clinical frailty by classifying older individuals based on vulnerability and risk of death, using simple and practical clinical criteria. Initially proposed to predict early mortality in elderly patients admitted to emergency departments, it was later extended to other medical fields, including oncology, but it has never been specifically validated in AML [27, 28, 29]. Interestingly, our study demonstrated that a higher frailty score assessed by the CSHA CFS was associated with worse survival, lower response rate, and a higher rate of infections. No correlation, however, was found with the occurrence of other TRAEs. As expected, patients with a frailty score > 3 experienced more hospitalizations due to TRAEs, reflecting not only the higher frequency of TRAEs but also the more challenging management of complications in frail individuals outside the hospital setting. Interestingly, in our analysis a higher CSF score did not correlate with a higher CCI score, highlighting that these two tools assess different clinical dimensions. Notably, in our analysis a higher CCI was not predictive of poorer survival. This finding can be explained by the fact that CCI focuses exclusively on predefined comorbidities, whereas leukemic patients may be considered frail for other reasons—such as pre‐treatment infections or reduced functional capacity—that are not captured by the CCI.

In our study the survival was influenced not only by frailty score but also by the type of HMAs used. Patients treated with azacitidine plus venetoclax achieved better survival compared with those treated with decitabine plus venetoclax (*p* = 0.025). To date, no randomized clinical trial has directly compared the two HMAs in combination with venetoclax. Indirect evidence from elderly TN AML patients treated with HMAs alone has shown no difference in CR rates or OS between azacitidine and decitabine [32]. In a retrospective series of 57 relapsed/refractory AML patients treated with AZA/VEN (22) or DEC/VN (35), no survival difference was observed between the two groups [33]. These findings were confirmed by another retrospective study, where investigators reported higher mortality in patients with a high Charlson comorbidity index regardless of the HMA backbone [33]. Similarly, in a phase Ib trial testing different venetoclax doses with either azacitidine or decitabine, response rates were comparable, although survival was not directly compared [5]. When comparing AZA and DEC in patients with MDS, the treatment with AZA was associated with a significantly better survival and lower rate of severe cytopenia [34]. At present, ELN 2022 recommendations endorse the use of venetoclax in combination with an HMA for AML patients unfit for intensive therapy, without preference for one HMA over the other. Ongoing and future prospective comparative studies will help clarify whether one HMA backbone is superior.

Another important limitation of the present study is the inability to apply the ELN 2024 stratification, which is specifically designed for patients receiving low‐intensity regimens, due to the absence of next‐generation sequencing (NGS) data needed to obtain a complete genetic profile.

Nevertheless, several limitations should be acknowledged. The retrospective design introduces inherent biases, including selection bias and heterogeneity in supportive care practices. The sample size was relatively small, limiting the statistical power to detect rare toxicities or subtle efficacy differences. Finally, molecular profiling was incomplete in some patients, precluding analysis of outcomes in genetically defined subgroups.

In conclusion, despite some limitations, this study suggests that incorporating frailty assessment into routine clinical practice may help clinicians to better identify patients most likely to benefit from venetoclax‐based regimens, optimize supportive interventions, and avoid overtreatment in those at highest risk of complications. In such cases, HMA monotherapy or, in selected patients, other targeted therapies could represent safer options.

In our analysis, the AUC value of 0.75 indicates a fair to good discriminative ability of the model in identifying patients more likely to experience worse outcomes. Clinically, an AUC of 0.75 suggests that the model correctly ranks patients in 75% of cases, indicating its utility as a supportive tool. However, it should not be used as a standalone decision‐making instrument, but rather integrated with disease biology, comprehensive comorbidity assessment, and patient preferences to guide treatment choice.

Moreover, the CSF is a simple and practical clinical tool that can be applied not only at baseline but also throughout the treatment course, to guide potential dose reductions or treatment duration adjustments.

Broader validation of frailty tools in larger cohorts and prospective studies will be essential to refine patient selection and maximize the clinical utility of venetoclax–HMA therapy in elderly AML patients.

## Author Contributions

**Ernesto Vigna**, **Antonella Bruzzese** and **Massimo Gentile:** conceptualization. **Enrica Antonia Martino**, **Francesco Mendicino**, **Ernesto Vigna**, **Antonella Bruzzese**, and **Fortunato Morabito:** methodology. **Ernesto Vigna:** writing – Original draft preparation. **Ernesto Vigna**, **Antonella Bruzzese**, **Massimo Gentile**, and **Fortunato Morabito:** writing, review, and editing. All authors have read and agreed to the published version of the manuscript.

## Ethics Statement

The study has been approved by the ethics committee.

## Conflicts of Interest

The authors declare no conflicts of interest.

## Supporting information


**Table S1:** Hospitalization risk for TRAEs.
**Table S2:** Response to therapy.

## Data Availability

The data that support the findings are available from the corresponding author upon reasonable request.
