# Supplementary material for: Clinical Utility of Frailty Scoring in Elderly Acute Myeloid Leukemia Patients Treated With Venetoclax and Hypomethylating Agents
Source: Eur J Haematol. 2025 Oct 29;116(2):160–8. doi: 10.1111/ejh.70058 (PMC12781152; doi:10.1111/ejh.70058)
Supplement: Supplementary file 1 — Table S1: Hospitalization risk for TRAEs. Table S2: Response to therapy. [file EJH-116-160-s001.docx]

**Supplementary Table 1.** Hospitalization risk for TRAEs

|  | Patients with need of  hospitalization for TRAEs (%) | Patients without need of hospitalization for TRAEs (%) | P |
| --- | --- | --- | --- |
| Gender  Male  Female | 21 (63.6%)  8 (42.1%) | 12 (36.4%)  11 (57.9%) | 0.13 |
| Age (years)  <75  >75 | 13 (54.2)  16 (57.1) | 11 (45.8)  12 (42.9) | 0.83 |
| AML type  Primary  Secondary | 19 (57.6%)  10 (52.6%) | 14 (42.4%)  9 (47.4) | 0.73 |
| ELN  Favourable  Intermediate  Unfavourable  Not available | 2 (40%)  7 (63.6%)  12 (52.2%)  8 (61.5%) | 3 (60%)  4 (36.4%)  11 (47.8%)  5 (38.5%) | 0.78 |
| BMI  <25  > 25 | 21 (56.8%)  8 (53.3%) | 16 (43.2%)  7 (46.7%) | 0.82 |
| GFR (ml/min)  <30  >30 | 4 (50%)  25 (56.8%) | 4 (50%)  19 (43.2%) | 0.72 |
| Type of therapy  Aza/ven  Dec/ven | 20 (60.6%)  9 (47.4%) | 13 (39.4%)  10 (52.6%) | 0.35 |
| Frailty scale  <3  >3 | 15 (78.9%)  14 (42.4%) | 4 (21.1%)  19 (57.6%) | **0.011** |
| CCI  <6  >6 | 22 (56.4%)  7 (53.8%) | 17 (43.6%)  6 (46.2%) | 0.87 |

**Legend**: ELN: European Leukemia Network; GRF: Glomerular Filtration Rate; BMI: body mass index; AZA: azacitidine; CCI: Charlson Comorbidity Index; DEC: decitabine.

**Supplementary Table 2. Response to therapy**

|  | > Partial remission | < Partial remission | P |
| --- | --- | --- | --- |
| Gender  Male  Female | 16 (48.5%)  9 (47.4%) | 17 (51.5%)  10 (52.6%) | 0.94 |
| Age (years)  <75  >75 | 10 (41.7)  15 (53.6) | 14 (58.3)  13 (46.4) | 0.39 |
| AML type  Primary  Secondary | 17 (51.5%)  8 (42.1%) | 16 (48.5%)  11 (57.9%) | 0.51 |
| ELN 2017  Favourable  Intermediate  Unfavourable  Not available | 3 (60%)  6 (64.5%)  9 (39.1%)  7 (53.8%) | 2 (40%)  5 (45.5%)  14 (60.9%)  6 (46.2%) | 0.71 |
| BMI  <25  >25 | 21 (56.8%)  4 (33.3%) | 16 (43.2%)  11 (66.7%) | 0.07 |
| GFR (ml/min)  <30  >30 | 2 (25%)  23 (52.3%) | 6 (75%)  21 (47.7%) | 0.15 |
| Type of therapy  Aza/ven  Dec/ven | 17 (51.5%)  8 (42.1%) | 16 (48.5%)  11 (57.9%) | 0.51 |
| Frailty scale  <3  >3 | 14 (73.7%)  11 (33.3%) | 5 (26.3%)  22 (66.7%) | **0.005** |
| CCI  <6  >6 | 21 (53.8%)  4 (30.7%) | 18 (46.2%)  9 (69.3%) | 0.15 |

**Legend**: ELN: European Leukemia Network; GFR: Glomerular Filtration Rate; BMI: body mass index; AZA: azacitidine; CCI: Charlson Comorbidity Index; DEC: decitabine.
